# Preventive effect of taurine on experimental type II diabetic nephropathy 

**DOI:** 10.1186/1423-0127-17-S1-S46

**Published:** 2010-08-24

**Authors:** Shumei Lin, Jiancheng Yang, Gaofeng Wu, Mei Liu, Xinhong Luan, Qiufeng Lv, He Zhao, Jianmin Hu

**Affiliations:** 1 College of Animal Science & Veterinary Medicine, Shenyang Agricultural University, Shenyang, 110866, P.R. China

## Abstract

**Background:**

It has been verified that taurine has some preventive effects on diabetes and its complications when used alone or together with other drugs, but there are few reports about taurine on the prevention of diabetic nephropathy, the mechanisms of which are still unknown.

**Methods:**

Taurine was administered to type Ⅱ diabetic rats induced by high fat high sugar diet combined with STZ injection. The preventive effect of taurine on diabetic nephropathy was investigated by detecting blood glucose, lipid metabolism, kidney function and glomerular basement membrane metabolism.

**Results:**

Taurine could lower blood glucose, TG, TC, BUN, Scr, NAG, U-PRO, the expression of laminin B1( LBN1) mRNA, and increase HDL-C of diabetic rats.

**Conclusions:**

The results indicated that taurine could prevent the occurrence and development of diabetic nephropathy by decreasing blood glucose, improving lipid metabolism, glomerular basement membrane metabolism, and kidney function.

## Introduction

Diabetic nephropathy (DN) whose incidence is up to 47.66% is the most common and difficult diabetic microvascular complication to treat and has become the first cause of end-stage renal disease [1-3]. It is reported that about 43% of the chronic renal failure (CRF) patients on dialysis are DN, 60% case fatality of diabetes mellitus (DM) patients are DN, DM patients who died of renal failure are 17 times more than non-DM patients [4]. Therefore, prevention of the occurrence and the development of DN has become a very urgent issue.

Taurine, a sulfur-containing β-amino acid with a wide range of biological effects, is the most abundant free intracellular amino acid presents in many tissues of humans and animals [5]. Researches have demonstrated that taurine has some preventive and curative effects on DN. H.Trachtman (1995) found that 1% taurine supplementation in drinking water for 52 weeks could reduce total proteinuria (U-PRO) and albuminuria by nearly 50%. This treatment also prevented glomerular hypertrophy, diminished glomerulosclerosis and tubulointerstitial fibrosis in diabetic animals [6]. It was reported by A.Erden (2000) that taurine could reduce gentamicin induced increases in serum creatinine (Scr), 24h urine volume, serum urea nitrogen (BUN) and tissue lactate and TBARS levels [7]. S.Higo (2008) reported that taurine administration significantly suppressed further increase in urinary protein excretion in diabetic rats [8]. Four weeks after intravenous injection of 50 mg/kg streptozotocin (STZ), diabetic rats exhibited 6.1 fold increase in urinary protein excretion, taurine supplement by 1% in drinking water prevented increases proteinuria [9].The reports mentioned above indicated that the previous study about taurine on DN were all concerned with typeⅠDN induced by STZ. There were no reports about preventive effects of taurine on type Ⅱ DN by artificial induction. In diabetes mellitus, expansion of the glomerular mesangium correlates with the clinical features of diabetic kidney disease. The increase in mesangial matrices is due primarily to the accumulation of normal matrix proteins, including collagens type IV and type V, laminin (LN), and fibronectin [10]. As a main protein composition in glomerular basement membrane, LN could reflect the dynamic state of extracellular matrix (ECM) synthesis. So LN was considered as a main index for renal injury, as well as for the development of DN. There were no reports about the effect of taurine on LN.

In this study, the preventive effect and its mechanism of taurine on DN would be investigated, in order to provide a theoretical basis for clinical application of taurine.

## Methods

### Experimental animals and treatments

Six-week-old male Wistar rats weighing 140-180g were maintained under a controlled condition of light (12h of light,12h of dark) and temperature (23±2℃), and were given free access to food (commercial standard rat chow) and water.

One hundred and ten male Wistar rats weighing 140-180g were randomly divided into two groups: normal control group (A group, 20 rats) and model group (M group, 90 rats). Rats in M group were fed with a high sugar, high fat diet for one month to induce insulin resistance (IR), and then injected with STZ (25mg/kg) once per week for two weeks. Rats in A group were injected with citric acid-citrate sodium buffer solution. Rats in M group were fed with a high sugar, high fat diet for two months after STZ injection. Then fasting blood glucose, random blood glucose and fasting insulin were detected seven days after the second STZ injection. DM rats in M group were divided into four groups randomly (18 rats in each group): spontaneous recovery group (B), high concentration of taurine group (C), medium concentration of taurine group (D) and low concentration of taurine group (E). Rats in B, C, D and E groups were administered with 0mg/kg, 3.4mg/kg, 2.6mg/kg, and 2.1mg/kg 70% taurine suspension respectively. Rats in A and B groups received the same treatment. At the end of the 6th and the 10th weeks, five rats were selected from each group, blood was collected from the jugular vein, then BUN, Scr, β-N-acetyl-glucosaminidase (NAG), triglyceride (TG), total cholesterol (TC) and high-density lipoprotein cholesterol (HDL-C) were detected. Twenty-four h urine samples were collected and U-PRO was detected. Renal basement membrane adhesion protein expression was detected by way of in-situ hybridization.

### Chemicals

Blood glucose meters and blood glucose test strips with lot number were purchased from U.S. Roche. STZ was purchased from Sigma Chemical Company. Sugar, cholesterol, and bile salt were purchased from Shenyang Chemical Reagent Co., Ltd. Taurine was purchased from Beijing Capital Commercial Source Co., Ltd. Insulin RIA kit was purchased from China Institute of Atomic Energy Research Institute of Isotopes. BUN, Scr, NAG, U-PRO, TG, TC, HDL-C kits were purchased from Nanjing Jiancheng Bioengineering Institute. Diethyl pyrocarbonate (DEPC), Paraformaldehyde, Special coverslip in situ hybridization and in situ hybridization detection kits were purchased from Wuhan Boster Biological Engineering Co., Ltd.

### Experimental diets

Normal diet was purchased from Yuhong District test animal feed factory (Shenyang), the nutrition ingredient of which is in accordance with the nutritional standards. High-fat high-sugar feed was purchased from Yuhong District test animal feed factory (Shenyang) which contains 15% lard, 25% sucrose, 2.5% cholesterol, 1% bile salts and 56.5% normal feed.

### Judgments of model

Determination of fasting blood glucose: After an overnight (10–12 h) fast, during which only water was permitted. Blood was collected from the tail. The first drop of blood was wiped off, and the second drop of blood was used to determine the blood glucose using blood glucose test strips.

Determination of random blood glucose: without fasting, methods were the same as mentioned above. Determination of insulin levels: 200-300μL whole blood was collected from the tails. After standing at room temperature for 4h, serum was separated by centrifuging at 1500 rpm for 15 min at 4℃. INS was determined using an insulin RIA kit.

The establishment of animal models of T2DM must meet the following two conditions:

(1) ISI = Ln [1 / (fasting glucose × serum insulin level)] ≤the mean value of normal animals;

(2) the mean value of random blood glucose levels in normal rats +2 standard deviation ≤ the random blood glucose of model group.

### Standardization of insulin resistance

HOMA-IR=(FBGxFINS)／22.5 was used to evaluate the degree of insulin resistance, while ISI=1／(FBGxFINS) was used to demonstrate the insulin sensitivity. Calculate the natural logarithm of HOMA-IR and ISI [11].

### Biochemical analysis

Blood samples were collected from the jugular vein of 5 rats randomly selected from each group at the end of the 6th and the 10th weeks. After standing at room temperature for 4h, serum was separated by centrifuging at 1500 rpm for 15 min at 4℃, stored at -20 ℃. BUN, Scr, NAG, TG, TC, HDL-C were determined by colorimetry using kits. U-PRO was detected using urine collected from a metabolic cage for 24 hours.

### Kidney in-situ hybridization

The paraffin section of the rat’s kidney was dewaxed. Endogenous peroxidase was deactivated after being treated with 3% hydrogen peroxide at room temperature for 10 min. Then the section was digested with pepsin at 37 ℃ for 20 min in order to expose the mRNA nucleic acid fragment. Dropped 20μl oligonucleotide probe hybridization solution containing Cardiox was added on each slice over night. Then confining liquid, biotinylation rat anti biotinylation antibody and SABC were added to each section. They were then colored with DAB, redyed with hematoxylin, transparented with xylene, and mounted with neutral gum. The assessment criteria of positive cells: the cell is positive if there are brown granules in the cytoplasm observed under optical microscope. Negative control of in situ hybridization: 0.5mol/LPBS was used instead of nucleotide probe hybridization solution, repeat the above steps. The expression value was measured using the average relative gray scale.

### Statistical analysis

Data were presented as the mean ± SD and significant differences were determined by Duncan’s multiple range tests using SPSS 16.0 statistical analysis software. P values less than 0.05 were considered as significant, P values less than 0.01 were considered as extremely significant.

## Results

### Percentage of model establishment

Random blood glucose and ISI of groups A and M were shown in Table 1. The result meets the requirements of the T2DM rat model. 74 rat models were established, the percentage was 82%.

**Table 1:** Results of the random glucose level and ISI of Group C and M.

Group	amount	random blood glucose (mmol/L)	ISI
Model group	74	15.03±9.53	-5.13±0.69
Control group	20	6.3±0.46	-4.32±0.46

Results are presented as mean±SD C: control group, M: model group

Table 1 The results of the level of random glucose and ISI of Group A and M

### Insulin resistance analysis

FBG, FINS, ISI and HOMA-IR of groups A and M were shown in Table 2.The FBG and HOMA-IR of Group M was significantly higher (P ＜0.01) than the normal rats at the seventh day after STZ injection. The FINS and ISI of Group M was significantly higher (P  ＜0.05) than the normal rats. The results indicated that 74 rats in the model group all exhibited insulin resistance, which means that the rats were type Ⅱ diabetes models.

**Table 2:** Results of the insulin resistance of Group C and M

Group	amount	FBG (mmol/L)	FINS(IU/L)	ISI	HOMA-IR
Model group	74	5.43±0.75^**^	34.59±13.86^*^	-5.13±0.69^*^	2.04±0.40^**^
Control group	20	4.02±0.29	21.45±5.33	-4.32±0.46	1.36±0.26

Results are presented as mean±SD C: control group, M: model group

Table 2 The results of the insulin resistence of Group A and M

^*^P ＜0.05      ^**^P＜0.01

### Blood glucose analysis

**Figure 1:**
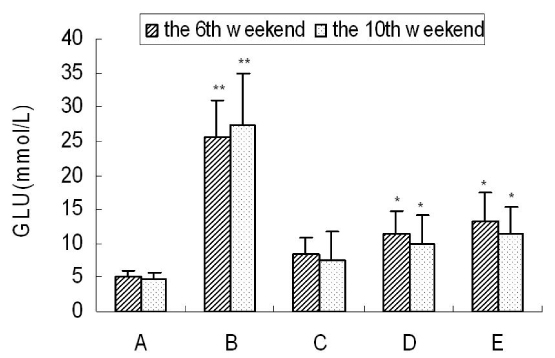
**Levels of GLU in each group at the 6th and the 10th weekends.** Results are presented as mean±SD (n=5). *: significantly different from control group (p<0.05), **: extremely significantly different from control group (p<0.01).

Figure 1 showed that the blood glucose level of spontaneous recovery group was significantly higher (P ＜0.01) than the normal rats at the end of the 6th and the 10th weeks. The blood glucose level of the taurine- preventive groups was significantly lower than that of the spontaneous recovery group, and particularly the high-concentration of taurine group had no significant differences compared with the control group.

### Analysis of lipid metabolism indexes

**Figure 2:**
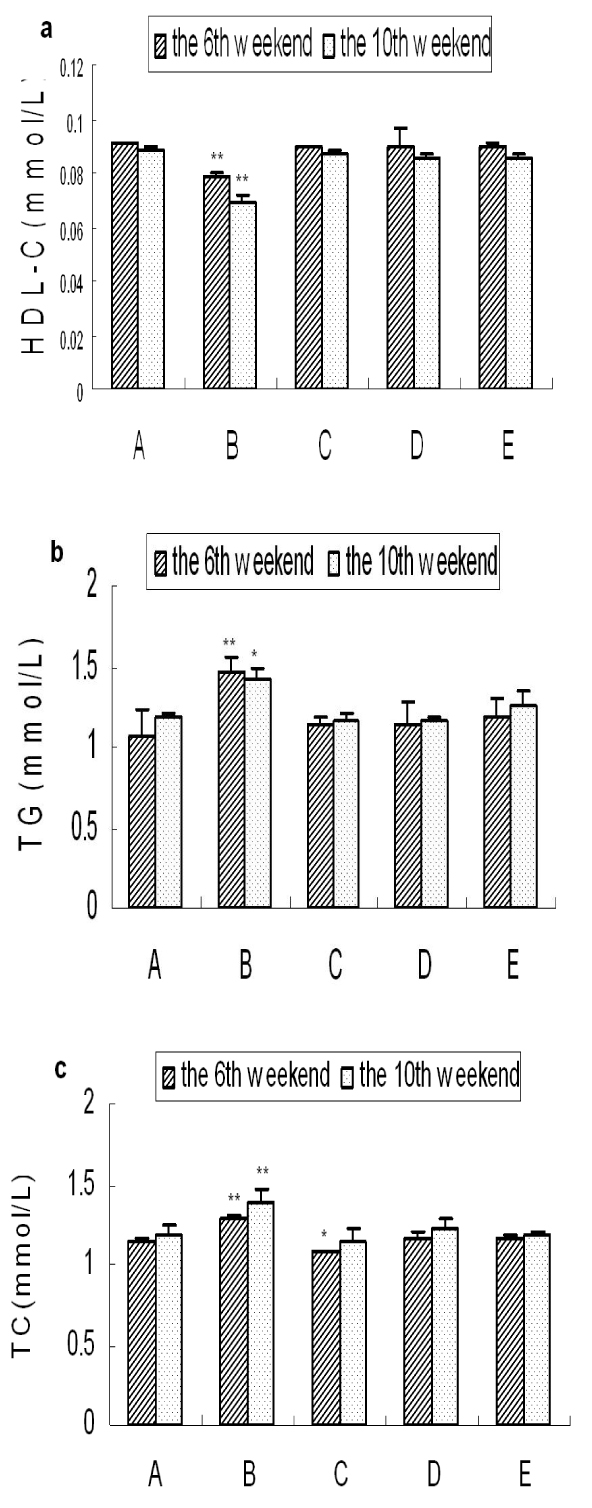
**Levels of lipid metabolism indexes in each group.** a: The level of HDL-C, b: The level of TG, c: The level of TC,. Results are presented as mean±SD (n=5). *: significantly different from control group (p<0.05), **: extremely significantly different from control group (p<0.01).

As shown in Fig. 2, serum concentrations of HDL-C, TG and TC in the spontaneous recovery group were significantly affected compared with the control group (P＜0.01 or P＜0.05), among which, the concentration of HDL-C was significantly decreased, and the concentrations of TG and TC were significantly increased. Taurine could significantly improve the indexes associated with lipid metabolism nearly to the normal levels.

### Analysis of kidney functional parameter

**Figure 3:**
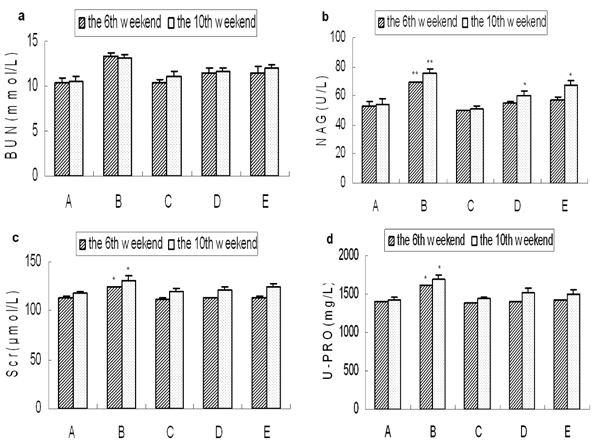
**Levels of kidney functional parameter.** a: The level of BUN, b: The level of NAG, c: The level of Scr, d: The level of U-PRO. Results are presented as mean±SD (n=5). *: significantly different from control group (p<0.05), **: extremely significantly different from control group (p<0.01).

As shown in Fig. 3, the concentrations of BUN, Scr and NAG were significantly higher in the spontaneous recovery group compared with the control group, while there were no significant differences between the taurine and the control groups. At the end of the 10th week, the concentration of NAG in the high concentration of tautine group was significantly lower than that of the control group, while the concentration of which was significantly higher than the control group.

### Expression of kidney basement membrane adherent protein

**Figure 4:**
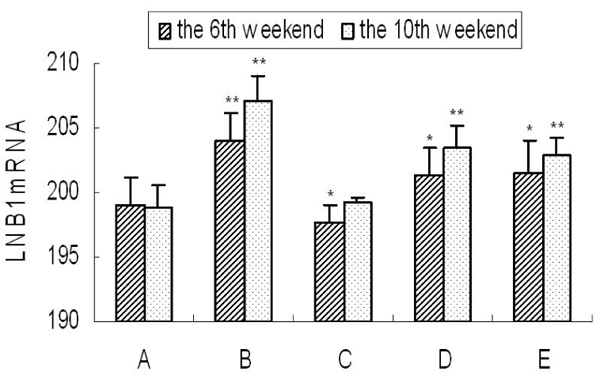
**Levels of kidney basement membrane adherent protein expression.** A: LNB1mRNA expression level of the control group At the end of the 6th and the 10th weeks, B: LNB1mRNA expression level of the spontaneous recovery group At the end of the 6th and the 10th weeks, C:LNB1mRNA expression level of high concentration of taurine group At the end of the 6th and the 10th weeks, D: LNB1mRNA expression level of the medium concentration of taurine group At the end of the 6th and the 10th weeks, E: LNB1mRNA expression level of the low concentration of taurine group At the end of the 6th and the 10th weeks. Results are presented as mean±SD (n=4). *: significantly different from control group (p<0.05), **: extremely significantly different from control group (p<0.01).

As shown in Fig. 4, the expression level of LNB1mRNA in the spontaneous recovery group was extremely significantly higher than the control group (P<0.01). At the end of the 6th week, the expression levels in the low and medium concentration of taurine groups were significantly higher than the control group (P<0.05), while which in the high concentration of taurine group was significantly lower than the control group (P<0.05). At the 10th weekend, the expression levels in the medium and the low concentration of taurine groups were extremely significant higher than the control group (P<0.01), while the high concentration of taurine group had no significant differences compared with the control group.

**Figure 5:**
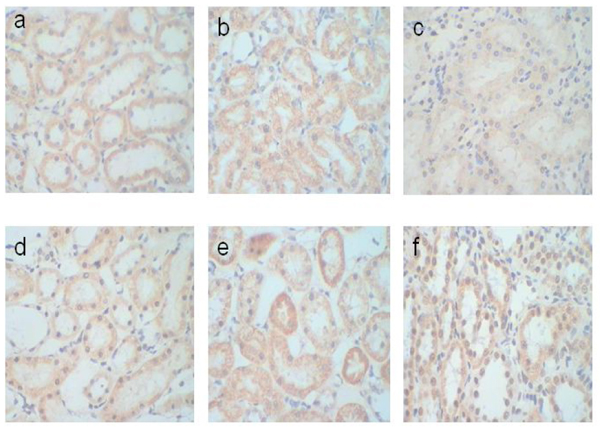
**In situ hybridization of adherent protein expression in kidney basement membrane.** The brown area was the positive expression area, enlarge 400ⅹ under light microscope. a: LNB1mRNA expression level of the control group at the end of the 6^th^ week，b: LNB1mRNA expression level of the spontaneous recovery group at the end of the 6^th^ week，c: LNB1mRNA expression level of the high concentration of taurine group at the end of the 6^th^ week， d: LNB1mRNA expression level of the control group at the end of the 10^th^ week，e：LNB1mRNA expression level of the spontaneous recovery group at the end of the 10^th^ week，f: LNB1mRNA expression level of the high concentration of taurine group at the end of the 10^th^ week.

It was shown in Fig. 5 that at the end of the 6th and the 10th weeks, the expression level of LNB1mRNA in the spontaneous recovery group was significantly higher than the control group. At the end of the 6th week, the expression level in the high concentration of taurine group was significantly lower than the control group.At the end of the 10th week, the expression level in the high dose of taurine group had no significant differences compared with the control group, meanwhile it was lower than the spontaneous recovery group at the end of the 6th and the 10th weeks.

## Discussion

### Blood glucose level and DN

In 1993, a report from American Diabetes Academy and the diabetes control and complications trial（DCCT）published in the New England Medical Journal verified that intensification therapy of insulin could keep the blood glucose at normal level and postpone the occurrence and the development of DN [12]. In addition, a perspective study in England verified that intensification therapy of insulin could obviously decrease the occurrence of type Ⅱ DN [13]. These clinical researchers demonstrated the effect of high glucose in the occurrence and the development of DN. It has been reported that taurine administered to STZ induced diabetic ddy or CD-1 mice could not only decrease the high blood glucose level, but also inhibit the decrease of serum insulin [14]. Harold OG et al and Donadio et al found that taurine preventively given to STZ induced insulin-dependent DM animal models could significantly decrease blood glucose, increase glucose content in skeletal and cardiac muscles, facilitate the composition and degradation of glycogen, and promote the transformation from 3H-glucose to glycogen [15,16]. But Ha et al (1999) believed that taurine could not decrease blood glucose. This study confirmed that taurine could effectively reduce blood glucose, prevent the occurrence and development of DN, which is consistent with most reports.

### Lipid metabolism and DN

Clinical research found that lipid metabolism disorder is an independent risk factor of type ⅡDN. It has been confirmed by animal experiment that hypercholesteremia could cause focal glomerulosclerosis. Kasiske et al. have reported that hypertriglyceridemia accelerated the occurrence of albuminuria and glomcrulus damage in obesity model rats [17]. The increase of blood is equal to the increase of urinary albumin excretion rate. The decrease of the blood fat could protect the kidney from glomerular sclerosis.

HDL-C is a protective factor against atherosclerosis. It can prevent the occurrence of renal artery atherosclerosis, reducing the probability of DN. Lin (1998) and Zhao (2005) confirmed that taurine could decrease serum TG, TC, increase HDL-C level of rats fed with high fat diet, which means that taurine could improve lipid metabolism of rats fed with high fat diet [18,19]. Nishimura investigated rats with genetic type ⅡDM and found that taurine could obviously decrease plasma TC in rats fed with high cholesterol diet. While taurine had no effect on plasma TC in rats fed with normal diet, but could significantly increase plasma HDL-C in GK rats. These results indicated that taurine had a significant effect on decreasing blood fat and promoting lipid metabolism. Murakami et al administered taurine in drinking water and found that taurine could obviously decrease plasma TC and increase plasma HDL-C in rats [20]. This study showed that taurine could increase HDL-C, decrease TC and TG, which confirmed that taurine can reduce the probability of DN by effectively improving lipid metabolism.

### Basement membrane adherent protein expression and DN

The main pathological changes of DN is the thickening of the glomerular capillary basement membrane, the proliferation of mesangial cells and the increase of mesangial matrix, the thickening of tubular basement membrane, and eventually leading to diffuse or nodular glomerulosclerosis, and tubulointerstitial interstitial fibrosis. LN which located inside the loose layer of GBM, is an important constitution of GBM and ECM. LN plays important roles in mediating the interaction between the cells and matrix, cell adhesion, migration, proliferation and differentiation. LN can cause cell adhesion and affect the selectivity of GBM charge. As a sign of kidney damage, LN is an important index for DN development [21]. Immunohistochemically an increase of LN, collagen III and IV staining was observed in the mesangium and in the glomerular basement membrane [22]. The first laminin was isolated in 1979 from the mouse Engelbreth–Holm–Swarm (EHS) tumour [23]. LN is a large basement membrane-specific glycoprotein composed of three chains:A,B1,B2 [24]. Structural analysis using electron microscopy revealed LN as a cruciform-like structure with three short arms and one long arm [25]. The long arm consists of an α-helical coiled coil and is the only region in which all three chains associate [26]. In fact, in the absence of collagen IV, the other major component that assembles into a scaffold, LN has been shown to be sufficient for forming basement membrane-like matrices during early development of mice embryos [27]. Immunostaining for laminin was increased in the diabetic male SD rats (200–250g) by intravenous injection of STZ in citrate buffer (50 mg/kg) as compared with the control group [28]. Light and electron microscopy demonstrated a moderate increase of mesangial matrix and thickening of the glomerular basement membrane in diabetic rats. Fukui et al. reported increased mRNA levels for α1(IV) collagen, LNB1 and B2, and α1(I) and α1(III) collagen in diabetic rats 4 wk after STZ before morphological thickening of basement membrane occurred [29]. LNB1 mRNA level was increased 1.7 fold in diabetic NOD mice than in agematched nondiabetic NOD mice with normal glucose tolerance [30]. In this experiment, LNB1 cDNA probes were used for in situ hybridization, which can efficiently detect the LN level in glomerular basement membrane. This study confirmed that high doses of taurine can effectively reduce the expression level of LNB1mRNA and improve the metabolism of glomerular basement membrane. However, this improvement was reduced with time, the specific mechanism of which remains to be further investigated.

## Conclusion

In conclusion, taurine has some preventive effect on experimental type Ⅱ DN, the mechanism of which may be due to the decreasing blood glucose , improving lipid metabolism, glomerular basement membrane metabolism, and kidney function.

## List of abbreviations used

DM: diabetes mellitus; DN: diabetic nephropathy; HDL-C: high-density lipoprotein cholesterol; TG: triglyceride; TC: total cholesterol; BUN: blood urea nitrogen; Scr: proteinuria; STZ: streptozotocin; DEPC: diethyl pyrocarbonate; ECM: extracellular matrix ; CRF: chronic renal failure; LN: laminin; LNB1: laminin B1; GBM: glomerular basement membrane; FBG: fasting blood glucose; FINS: fasting insulin; ISI: insulin sensitivity index; HOMA-IR: homeoestasis model assessment -insulin resistance.

## Competing interests

The authors declare that they have no competing interests.

## Authors' contributions

Shumei Lin carried out determination of blood glucose targets, participated in the study design and drafted the manuscript. Jiancheng Yang participated in the detection of insulin resistance, the design of the study and performed the statistical analysis. Gaofeng Wu carried out the Sampling and insulin determination. Mei Liu participated in detection of lipid metabolism. Xinhong Luan participated in detection of LNB1 in situ hybridization in kidney. Qiufeng Lv participated in rat raising. He Zhao carried out the establishment of type Ⅱ diabetes model. Jianmin Hu conceived of the study, and participated in its design and coordination and helped to draft the manuscript. All authors read and approved the final manuscript.
